# Case report: A hybrid open and endovascular approach for repairing a life-threatening innominate artery dissection using the simultaneous kissing stent technique

**DOI:** 10.3389/fneur.2023.1149236

**Published:** 2023-05-05

**Authors:** Chu-Hsuan Kuo, Shun-Tai Yang, Yueh-Hsun Lu, Yu-Chun Lu, I-Chang Su

**Affiliations:** ^1^Department of Primary Care Medicine, Shuang Ho Hospital, Taipei Medical University, New Taipei City, Taiwan; ^2^Department of Neurosurgery, Shuang Ho Hospital, Taipei Medical University, New Taipei City, Taiwan; ^3^Taipei Neuroscience Institute, Taipei Medical University, Taipei City, Taiwan; ^4^Department of Neurosurgery, School of Medicine, College of Medicine, Taipei Medical University, Taipei City, Taiwan; ^5^Department of Radiology, Shuang Ho Hospital, Taipei Medical University, New Taipei City, Taiwan; ^6^Department of Radiology, School of Medicine, College of Medicine, Taipei Medical University, Taipei City, Taiwan

**Keywords:** arterial dissection, hybrid approach, innominate artery, kissing stent, stenosis

## Abstract

Managing acute innominate artery (IA) dissection associated with severe stenosis is challenging due to its rarity, possible complex dissection patterns, and compromised blood flow to the brain and upper extremities. This report describes our treatment strategy for this challenging disease using the kissing stent technique. A 61-year-old man had worsening of an acute IA dissection secondary to an extension of a treated aortic dissection. Four possible treatment strategies for kissing stent placement were proposed based on different approaches (open surgical or endovascular) and accesses (trans-femoral, trans-brachial, or trans-carotid access). We chose to place two stents simultaneously via a percutaneous retrograde endovascular approach through the right brachial artery and a combined open surgical distal clamping of the common carotid artery with a retrograde endovascular approach through the carotid artery. This hybrid approach strategy highlights the three key points for maintaining safety and efficacy: (1) good guiding catheter support is obtainable through retrograde, rather than antegrade, access to the lesion, (2) concomitant cerebral and upper extremity reperfusion is guaranteed by placing kissing stents into the IA, and (3) peri-procedural cerebral emboli are prevented by surgical exposure of the common carotid artery with distal clamping.

## 1. Introduction

Acute symptomatic innominate artery (IA) dissection is challenging to manage when it presents with concomitant worsening of cerebral and upper extremity ischemia. Different revascularization methods, such as endovascular, open surgery, or combined approaches, have been proposed for IA dissection, but each has its pros and cons (1). Theoretically, the optimal treatment strategy in this emergency situation accomplishes three major goals: (1) timely recanalization of the IA, (2) reperfusion of the cerebral and upper extremities, and (3) cerebral embolization prevention during the revascularization procedure (1).

This case report discusses our treatment strategy for a patient with acute IA dissection associated with tight stenosis, complex dissection configuration, and emergency flow compromise.

## 2. Case description

**Figure 1:**
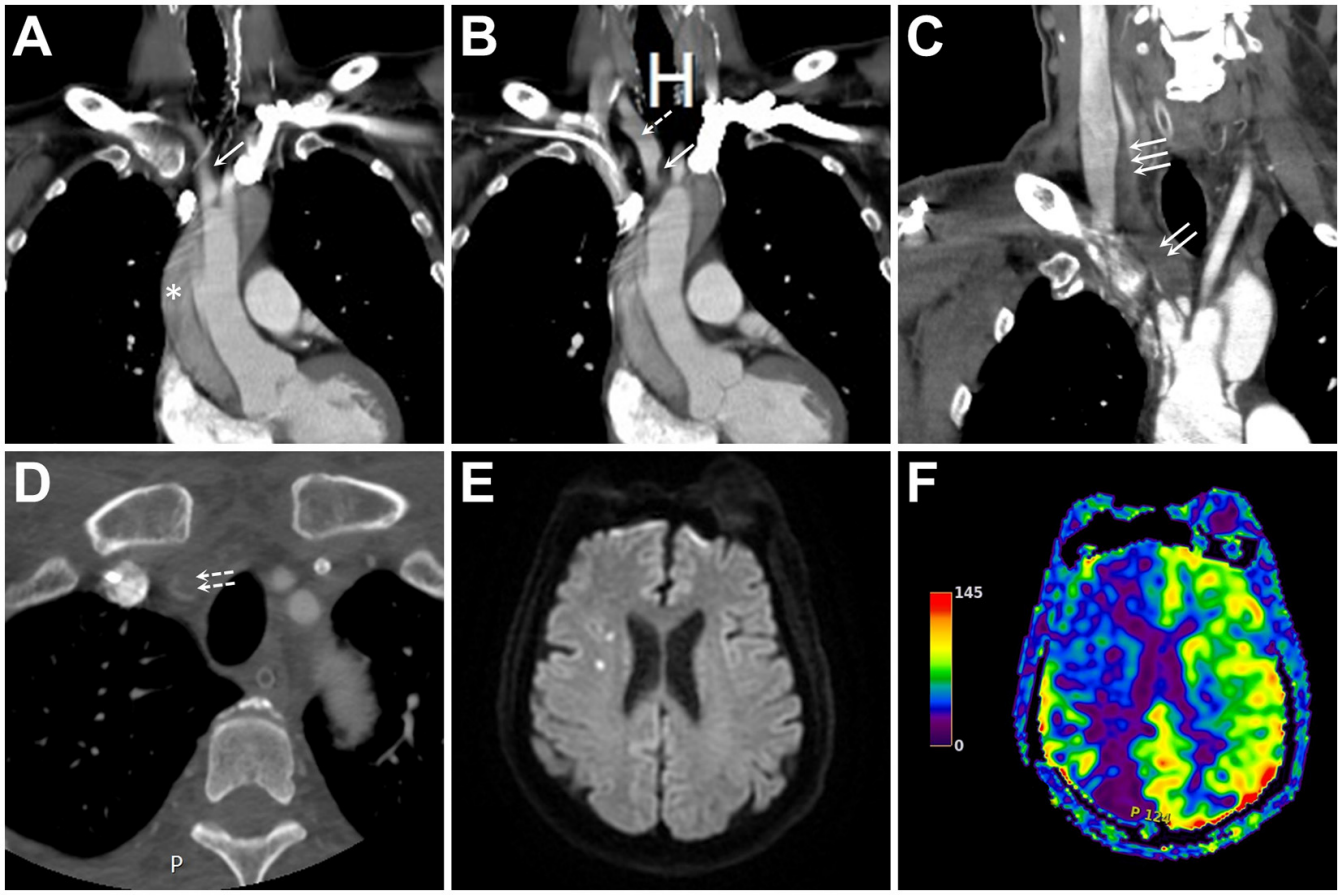
**(A, B)** Initial contrast-enhanced computed tomography (CECT) of the neck and chest in two consecutive coronal sections show an aortic dissection (asterisk) with innominate artery (IA) extension (single solid arrow). At this point, the main IA trunk remained patent without flow-limiting stenosis (single dashed arrow). **(C, D)** Coronal **(C)** and axial **(D)** images of CECT two days following ascending aortic grafting show expansion of the dissection's false lumen (double solid arrows), the distal extension of the dissection involving common carotid artery (triple solid arrows) and subclavian artery (double dashed arrows), and nearly complete occlusion of the main IA trunk and its bifurcation. **(E, F)** Diffusion-weighted imaging and arterial spin labeling perfusion magnetic resonance imaging of the brain reveal few embolic spots **(E)** associated with a large hypoperfusion in the right hemisphere **(F)**.

A 61-year-old male was admitted to our emergency department after the onset of sudden chest pain. The patient's neurological findings were normal, but their blood pressure was 92/72 mmHg without right and left differences. Furthermore, their pulse rate was 55 beats/min, and their respiratory rate was 22 breaths/min. Whole body contrast-enhanced computed tomography (CECT) revealed an aortic dissection extending from the ascending to the descending aorta, which also involved the IA and extended into the right common carotid artery (CCA) (Figures 1A, B). The patient, therefore, underwent emergency aortic graft placement for the ascending aorta.

The patient recovered well immediately after the operation. On postoperative day 2, the patient became lethargic, and his blood pressure in the right arm dropped to ~50/40 mmHg. CECT of the neck revealed a progression of the IA dissection, tight narrowing of the residual IA true lumen, and involvement of the right CCA and subclavian artery (SA) orifices (Figures 1C, D). Perfusion magnetic resonance imaging of the brain showed marked right-sided hemispheric hypoperfusion (Figures 1E, F).

**Figure 2:**
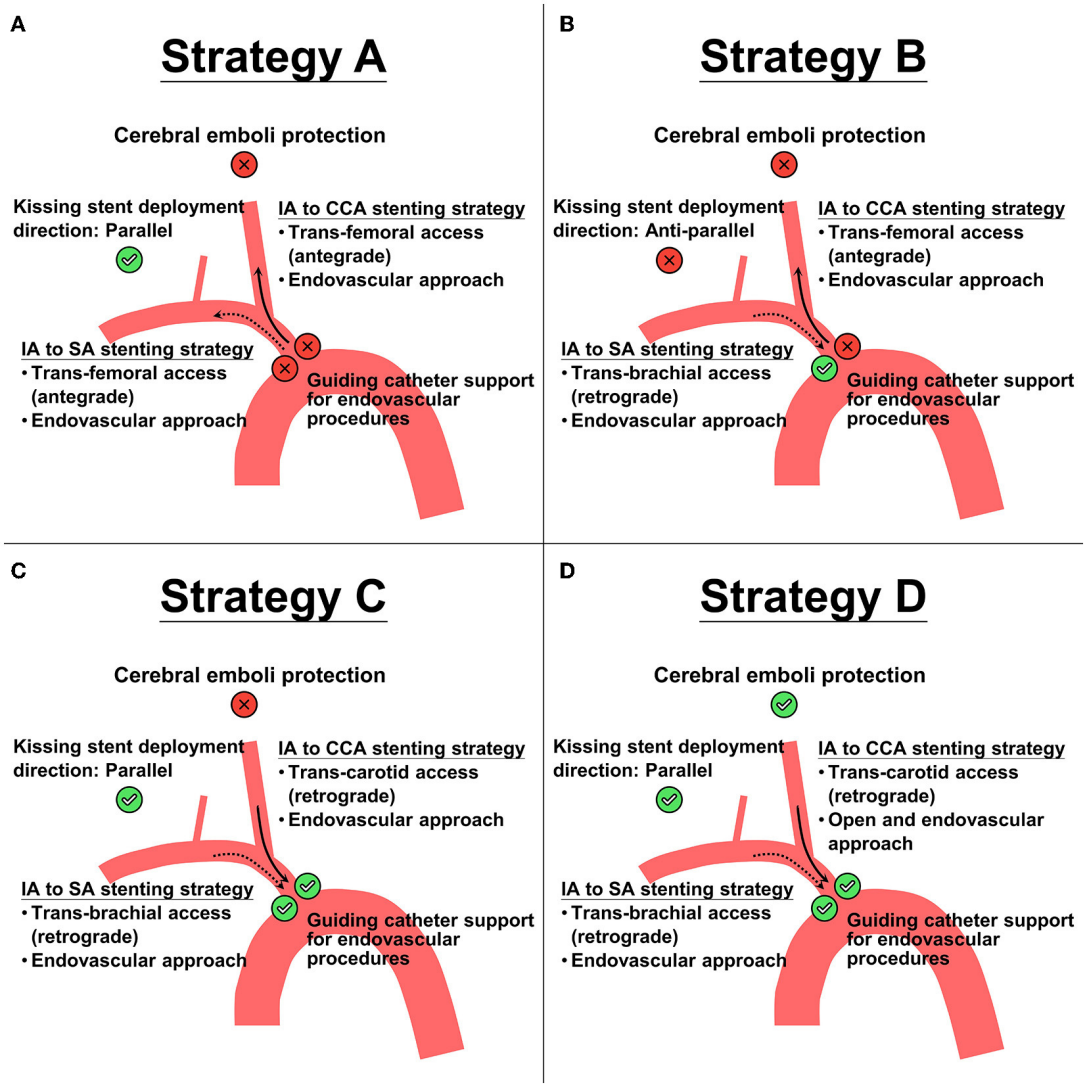
Four proposed treatment strategies **(A–D)** for kissing stent placements based on different accesses (trans-femoral, trans-brachial, or trans-carotid access) and different approaches (endovascular or a combined endovascular and open approach). Check-sign (with green circle background) and cross-sign (with red circle background) indicate the pros and cons, respectively, of the corresponding choice. Notice that strategy A and B have 1 pro and 3 cons, strategy C has 3 pros and 1 con, and strategy D has 4 pros and 0 cons. Solid arrow represents the direction of endovascular procedure in innominate artery (IA) to common carotid artery (CCA) stenting strategy. Dashed arrow represents the direction of endovascular procedure in IA to subclavian (SA) stenting strategy.

To obtain cerebral and upper extremity reperfusion, we planned to reconstruct the IA bifurcation using a simultaneous kissing stent technique. After comparing the pros and cons of four kissing stent reconstruction strategies (Figure 2) designed based on different approaches (endovascular or a combined endovascular and open approach) and different accesses (trans-femoral, trans-brachial, or trans-carotid access), we chose a combined open and endovascular approach (Strategy D in Figure 2) for the lesion because it could provide better guiding catheter support for the endovascular procedures and prevent peri-procedural cerebral emboli. As for the stent selection, we chose two self-expanding closed-cell stents of the same size, in order to provide an appropriate contact with the vessel wall, better coverage of the atheromatous lesions, and to minimize stent compromise by another stent during deployment. Based on the pre-treatment CECT, the diameters of IA, proximal CCA, and proximal SA were 15.1 mm (residual true lumen: 1.5 mm), 7.9 mm (residual true lumen: 1.9 mm), and 6.8 mm, respectively; in order to reduce the squeezing pressure on the dissected vessel, we chose stent size slightly smaller than the maximal CCA diameter, i.e., two 7 × 50-mm Wallstents (Boston Scientific), for IA reconstruction. This stent size suited the diameter of proximal SA (6.8 mm), and the overall diameter of the resulting kissing stents (i.e., 14 mm) was also appropriate for the dissected IA segment.

**Figure 3:**
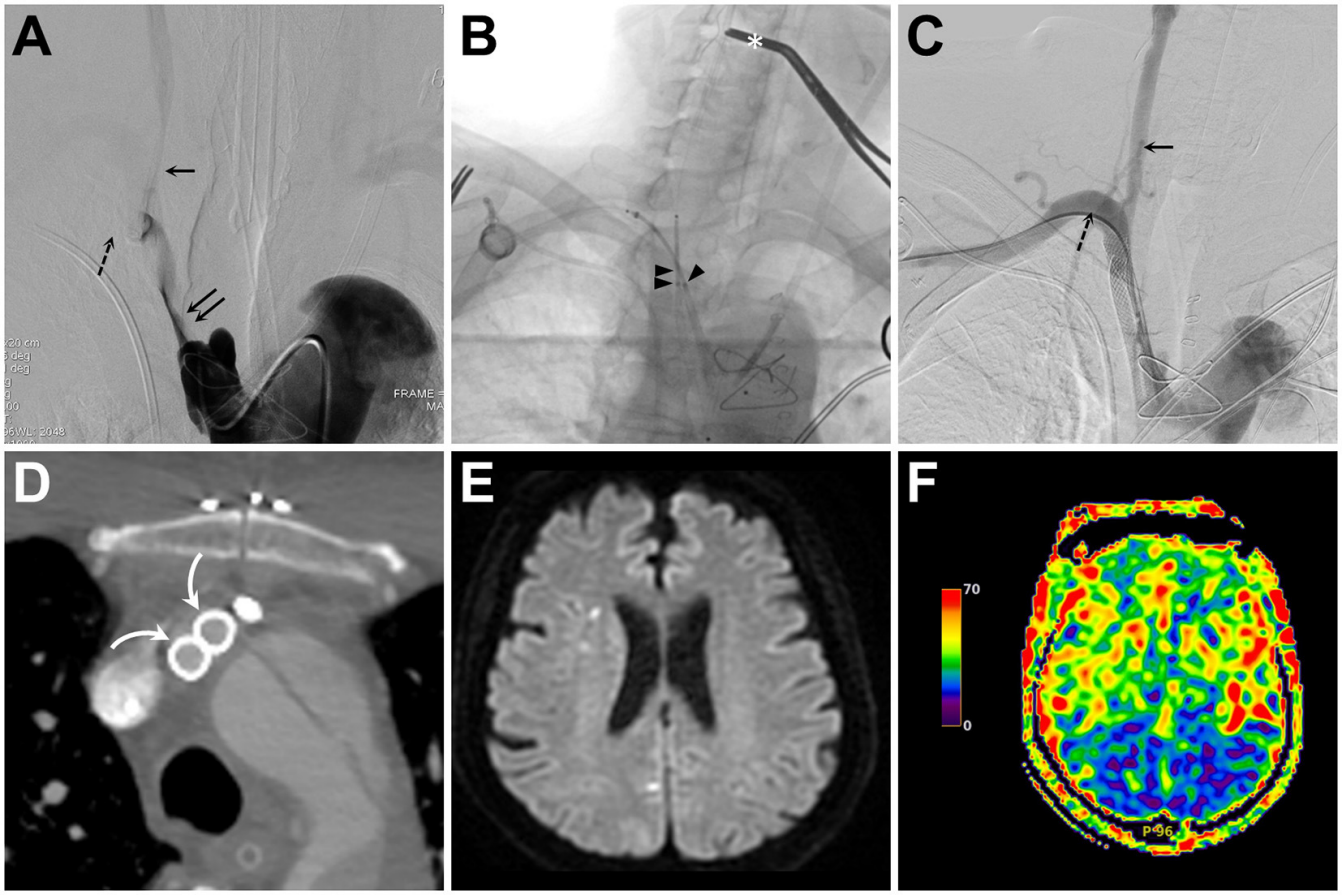
**(A)** Intra-innominate artery (IA) angiogram obtained from a transfemoral-placed angiocatheter reveals tight stenosis of the IA true lumen (double solid arrows) and compromised flow to the common carotid artery (CCA, single solid arrow) and subclavian artery (SA, single dashed arrow). **(B)** Fluoroscopic snapshot of the middle part of the kissing stenting procedure reveals simultaneous retrograde deployment of two Wallstents via a trans-brachial (single arrowhead) and a trans-carotid (double arrowheads) access. Also, notice the distal CCA clamp (asterisk) used for cerebral embolic protection during balloon angioplasty and stent deployment. **(C, D)** Following IA recanalization, both catheter angiography **(C)** and axial contrast-enhanced computed tomography **(D)** confirm good opening and apposition of both stents [curved arrow in **(D)**] and successful CCA (solid arrow) and SA (dashed arrow) recanalization **(C)**. **(E, F)** Diffusion-weighted imaging and arterial spin labeling perfusion magnetic resonance imaging of the brain show no increase in embolic spots **(E)** and symmetric hemispheric perfusion **(F)**.

The whole procedure was performed in the hybrid operating room equipped with a biplane angiography system (Artis Q biplane Siemens, Erlangen, Germany). The patient was loaded with 300 mg of aspirin and 300 mg of clopidogrel before the procedure. A standard surgical approach to the right carotid bifurcation was performed by making an incision and dissecting along the medial aspect of the right sternocleidomastoid muscle. A roadmap of the target lesion, together with the CCA and SA, was obtained using a 5F H1 diagnostic angiocatheter placed via trans-femoral access, with its tip in the IA orifice (Figure 3A). An 18-gauge needle was used to retrogradely puncture the exposed CCA, followed by the insertion of a 10 cm 8 French sheath. Meanwhile, a 6F Neuron 088 sheath was also retrogradely placed within the right subclavian artery via the trans-brachial access. Through each guiding sheath, a 300-cm 0.014” guidewire was navigated through the tight stenosis within IA to reach the descending aorta. With the distal CCA clamped, balloon dilatation at the IA was performed using an over-the-wire 12-mm Mustang dilating balloon (Boston Scientific, Marlborough, MA, USA), which was retrogradely advanced through the wire at the CCA. Through both microwires, two 7 × 50-mm Wallstents (Boston Scientific) were then simultaneously deployed in parallel from the IA to CCA and from the IA to SA (Figure 3B). Two 7-mm Sterling balloons (Boston Scientific) were then simultaneously used to post-dilate the deployed stents. After confirming the proper stent opening, the CCA sheath was removed, and all potential debris within the CCA was expelled by flushing the CCA through the puncture arteriotomy with heparinized saline. The arteriotomy was closed with a 6–0 Prolene suture, and the distal CCA clamp was removed. A final angiography and CECT showed the patent stents and successful CCA and SA reperfusion (Figures 3C, D).

The postoperative systolic blood pressure of the right upper extremity returned to normal. The patient also regained consciousness, and postoperative perfusion brain magnetic resonance imaging showed symmetric cerebral perfusion without new ischemic stroke (Figures 3E, F). The patient was treated with daily aspirin 100 mg and clopidogrel 75 mg for 3 months, followed by daily aspirin 100 mg. The patient remained neurologically intact, with a modified Rankin scale of 0, at 12 month follow-up.

## 3. Discussion

Ischemic stroke is one of the main issues after acute type A aortic dissection repair, with an incidence rate around 13% (2). Many factors are thought to be associated with this high ischemic stroke rate. One is the cerebral hypoperfusion secondary to the severe narrowing of the true lumen of the ascending aorta (3). The next common factor will be the progression of the dissection to the supra-aortic vessels including IA and CCA; in this situation, the mechanism of ischemic stroke can be explained by either emboli from thrombus within the false lumen or by a watershed damage secondary to the severe narrowing of the ICA or CCA (3). Therefore, in patients who received type A aortic dissection repair, CTA of both supra-aortic, extracranial, and intracranial arteries should be investigated thoroughly when ischemic stroke occurs, in order to delineate the possible ischemic etiology. As demonstrated in our patient, the mechanism of acute cerebral ischemia was hypoperfusion secondary to the severe narrowing of the IA dissection, and further surgical or endovascular treatment is mandatory because IA dissection progresses and even compromises the cerebral and upper extremity flow.

Before deciding on a treatment strategy, several challenges or key points regarding the recanalization of severe IA dissection should be considered. First, the IA is typically short in length and large in diameter. Thus, if the artery becomes severely narrowed, the trans-femoral guiding catheter can only be placed in the IA orifice, which is unstable and makes subsequent endovascular procedures difficult. In contrast, retrograde access via the CCA or SA can be confidentially obtained through the true lumen of the dissected IA, and it offers a stable position for the guiding catheter, reducing the risk of catheter dislodgement in the antegrade trans-femoral approach. In other words, endovascular retrograde access to the lesion is more favorable.

Second, the high thrombus burden within the large false lumen will inevitably increase embolic risks during any manipulation maneuver, such as repetitive catheterization, wiring, or balloon angioplasty. Previous literature clearly indicates that, although endovascular treatment of IA stenosis is associated with high technical success, it is also associated with 2–18% of post-interventional ischemic complications (1, 4). In other words, cerebral embolic protection is a key step in the strategic design for IA recanalization. As we chose not to pass the lesion via an antegrade trans-femoral approach, the embolic protection solution will be either endovascularly placing a protection device in the distal CCA via the brachial artery (5), or an open surgical approach for distal CCA clamping (1). We preferred the latter since embolic protection is achieved before any manipulations around the lesion are performed.

Finally, the treatment strategy should also aim to simultaneously preserve the cerebral and upper extremities during IA recanalization. This is crucial because dissection tears can be complicated, and the false lumen of IA dissection, as in our case, may involve the IA bifurcation and the CCA and SA. When balloon angioplasty or stenting is performed at the dissected IA via one of the IA branches, the thrombus may be squeezed, blocking the pathway from the IA to another IA branch (6). Therefore, we opted to adopt a simultaneous kissing stent technique to preserve both IA branches, which is widely used for treating arterial bifurcation lesions, such as aortoiliac occlusive diseases, or left main stem bifurcation coronary artery diseases (7, 8). This technique simultaneously deploys two parallel stents in the same direction to cover the main IA trunk and both IA branches. The important of choosing self-expanding stents and placing both stents in a “simultaneous and parallel” fashion cannot be overemphasized because this minimizes strut compromise by each other during deployment. Post-dilate both stents using two dilatation balloon of the same size in a synchronous inflation and deflation rate further guarantee that both stents will open better without compromise. In our patient, we specifically chose two self-expanding nitinol closed-cell stents of the same diameter for “kissing” because those stents are theoretically advantageous. For example, they provide even radial force for simultaneous expansion, decrease the chance of distal embolization owing to their superior ability to trap thrombotic materials, and reduce radial mismatch areas (7, 9).

All the above considerations formed the basis of our treatment strategy design in Figure 2. To fulfill all the favorable requirements, we chose to recanalize the IA and thus obtain cerebral and upper extremity reperfusion (and provide cerebral embolization protection) by performing retrograde balloon angioplasty and deploying two kissing stents through a percutaneous puncture of the right brachial artery in addition to surgically exposing and puncturing the CCA under distal CCA clamping. Our patient had significant postoperative clinical and radiological improvements and no new embolic strokes following the revascularization procedure, supporting the feasibility of such a treatment strategy.

In conclusion, this case details our treatment strategy for a complex acute IA dissection with severe narrowing in an emergency. Furthermore, we highlight the advantages of a hybrid open and endovascular approach for placing kissing stents for managing challenging IA dissections.

## Data availability statement

The original contributions presented in the study are included in the article, further inquiries can be directed to the corresponding author.

## Ethics statement

The studies involving human participants were reviewed and approved by TMU-Joint Institutional Review Board. Written informed consent for participation was not required for this study in accordance with the national legislation and the institutional requirements. Written informed consent was obtained from the participant/patient(s) for the publication of this case report.

## Author contributions

C-HK wrote the first draft of the manuscript. S-TY, Y-HL, Y-CL, and I-CS performed the procedure and revised the manuscript. All authors read and approved the final manuscript.
